# Synergistic actions of corticosterone and BDNF on rat hippocampal LTP

**DOI:** 10.1186/s13041-025-01213-x

**Published:** 2025-05-12

**Authors:** Jonathan S. Thacker, Liam T. Ralph, Laura Koek, Aram Abbasian, Luis B. Bettio, Ashleigh E. Smith, John Georgiou, Brian R. Christie, Graham L. Collingridge

**Affiliations:** 1 Lunenfeld-Tanenbaum Research Institute, Mount Sinai Hospital, Sinai Health, Toronto, ON M5G 1X5 Canada; 2 Tanz Centre for Research in Neurodegenerative Diseases, Temerty Faculty of Medicine, University of Toronto, Toronto, ON Canada; 3 Department of Physiology, Temerty Faculty of Medicine, University of Toronto, Toronto, ON Canada; 4 Division of Medical Sciences, University of Victoria, Victoria, BC Canada; 5 Alliance for Research in Exercise Nutrition and Activity (ARENA) Research Group, Division of Health Sciences, University of South Australia, Adelaide, Australia; 6 Behaviour, Brain, and Body (BBB) Research Group, Division of Health Science, University of South Australia, Adelaide, Australia; 7 Island Medical Program, University of British Columbia, Victoria, BC Canada

## Abstract

Exercise evokes many physiological changes, including the release of hormones and growth factors that are known to improve cognition via unknown mechanisms. Here, we have compared the ability of two physiologically relevant factors, corticosterone (CORT) and brain-derived neurotrophic factor (BDNF), to affect long-term potentiation (LTP) in the hippocampus. Using a compressed theta-burst stimulation (cTBS) protocol, we found that CORT has no effect on LTP, BDNF enhances LTP and combined CORT + BDNF treatment results in significantly greater LTP. We also find that CORT + BDNF, but not either compound alone, results in phosphorylation of protein kinase A (PKA). These findings show that BDNF and CORT act synergistically to enhance LTP at these synapses, potentially via a PKA-dependent mechanism. Such a synergistic action could underlie the positive cognitive effects of exercise.

**Supplementary Information:**

The online version contains supplementary material available at 10.1186/s13041-025-01213-x.

## Introduction

Exercise enhances learning and memory under both normal and pathological conditions, making it a valuable therapeutic approach for many brain disorders. Understanding the underlying mechanisms is crucial to fully harnessing its potential. Long-term potentiation (LTP) of synaptic transmission is commonly used to investigate the molecular and synaptic basis of learning and memory. At CA3-CA1 synapses, LTP can be divided into two mechanistically distinct forms, termed LTP1 and LTP2, that are distinguished by their independence or dependence, respectively, on the activation of protein kinase A (PKA) and calcium-permeable AMPARs (CP-AMPARs) [1, 2]. LTP2 is also distinct from LTP1 in that it involves *de novo* protein synthesis and can trigger heterosynaptic plasticity [3]. As such, LTP2 is hypothesized to be the more persistent form of LTP, which is likely to play a pivotal role in the formation and maintenance of associative long-term memory. Both corticosterone (CORT) and brain-derived neurotrophic factor (BDNF) have been found to facilitate LTP at CA3-CA1 synapses [4, 5]. Since CORT and BDNF are both released during exercise and contain considerable converging downstream mechanistic partners [6, 7, 8], we compared the effects of their combined application with that of either CORT or BDNF alone on LTP induced by theta-burst stimulation (TBS). As a proxy for LTP2-like engagement we examined the phosphorylation status of PKA following CORT and/or BDNF treatment. We demonstrate a synergistic effect of CORT and BDNF (CORT + BDNF) that may contribute to the exercise-induced enhancement on learning and memory.

## Methods

Male and female Sprague-Dawley rats aged six weeks were used in experiments. All experiments followed the Canadian Council on Animal Care guidelines and were approved by University of Victoria, The Centre for Phenogenomics (TCP) and University Health Network (UHN) animal care committees. Briefly, while under isoflurane anesthesia, brains were extracted into chilled, oxygenated artificial cerebrospinal fluid (ACSF), and 400 μm hippocampal coronal or transverse slices were sectioned using either a Compresstome^®^ VF-510-0Z (Precisionary Instruments LLC) or vibratome (VT1200S; Leica Biosystems), respectively. Slices were perfused with ACSF comprising (in mM): 125 NaCl, 2.5 KCl, 1.25 NaHPO_4_, 25 NaHCO_3_, 2 CaCl_2_, 1.3 MgCl_2_, 10 dextrose at pH = 7.3 and maintained at 30 °C. The field EPSPs were recorded from stratum radiatum in response to stimulation of Schaffer collateral-commissural (SCC) fibres. Baseline responses were obtained using a stimulus intensity set at approximately three times the threshold for evoking a fEPSP. LTP was induced by delivery of either tetanus (two trains of tetanus stimuli each 100 Hz, 1s; repeated after a 30s interval) or compressed theta-burst stimulation (cTBS) comprising three episodes of stimulation delivered 10 s apart. Each episode consisted of five bursts delivered at 5 Hz, with each burst comprising 5 stimuli at 100 Hz (75 stimuli in total). Slices were perfused with ACSF (vehicle (VEH))(containing BDNF ([20 ng/mL]; #B-250, Alomone Labs), CORT ([200 nM]; #27840, MilliporeSigma), or CORT + BDNF for 30 min prior to cTBS induction. The doses of 200 nM of CORT and 20 ng/mL of BDNF were chosen, as these concentrations are physiologically relevant and fall within the range of peripheral levels observed after acute exercise in humans [9, 10] and animals [8]. Furthermore, these doses have been shown in vitro to influence LTP induction without affecting resting membrane potential [5, 11, 12, 13]. We focused on a 30 min treatment window, as this duration corresponds to the exercise “dose” of our previous investigation [8], where we observed increases in CORT and BDNF, that were correlated to enhancement in plasticity-related phosphorylation.

We tested the effects of CORT, BDNF and CORT + BDNF on PKA activation as previously carried out on isolated tissue slices [7, 8]. Briefly, counterbalanced hippocampal slices (*n* = 8 slices/hemisphere) within animal from right and left hippocampus were randomly allocated to either VEH control or treatment. Slices were treated (30 min) in a BSK6 slice keeper (Scientific Systems Design Inc., Canada). Following bath application, all slices within condition were pooled (*n* = 8 slices/treatment per animal). Synaptoneurosome (SNP) fractions were prepared by the differential filtration method as previously described [7]. Western blotting was performed on 20 µg of hippocampal SNP to assess PKA phosphorylation (#5661S, Cell Signal Tech.), which was normalized to total PKA expression (#5842S, Cell Signal Tech.). Western blot was carried out using low fluorescence PVDF membranes blocked and incubated with primary and secondary antibodies (#12004162, #12005867, Bio-Rad Laboratories, Canada) and fluorescence intensity was captured on a ChemiDoc MP Imaging System (Bio-Rad Laboratories, Canada). Statistics were conducted (in GraphPad Prism) using one-way ANOVA with pre-planned Fisher LSD tests for LTP data whereas paired t-tests were used to evaluate treatment effects on PKA phosphorylation. All ANOVA results are corrected for multiple comparisons using Šidák correction and expressed as mean ± standard error. * = *p* < 0.05; *** = *p* < 0.001; **** = *p* < 0.0001.

## Results

**Fig. 1:**
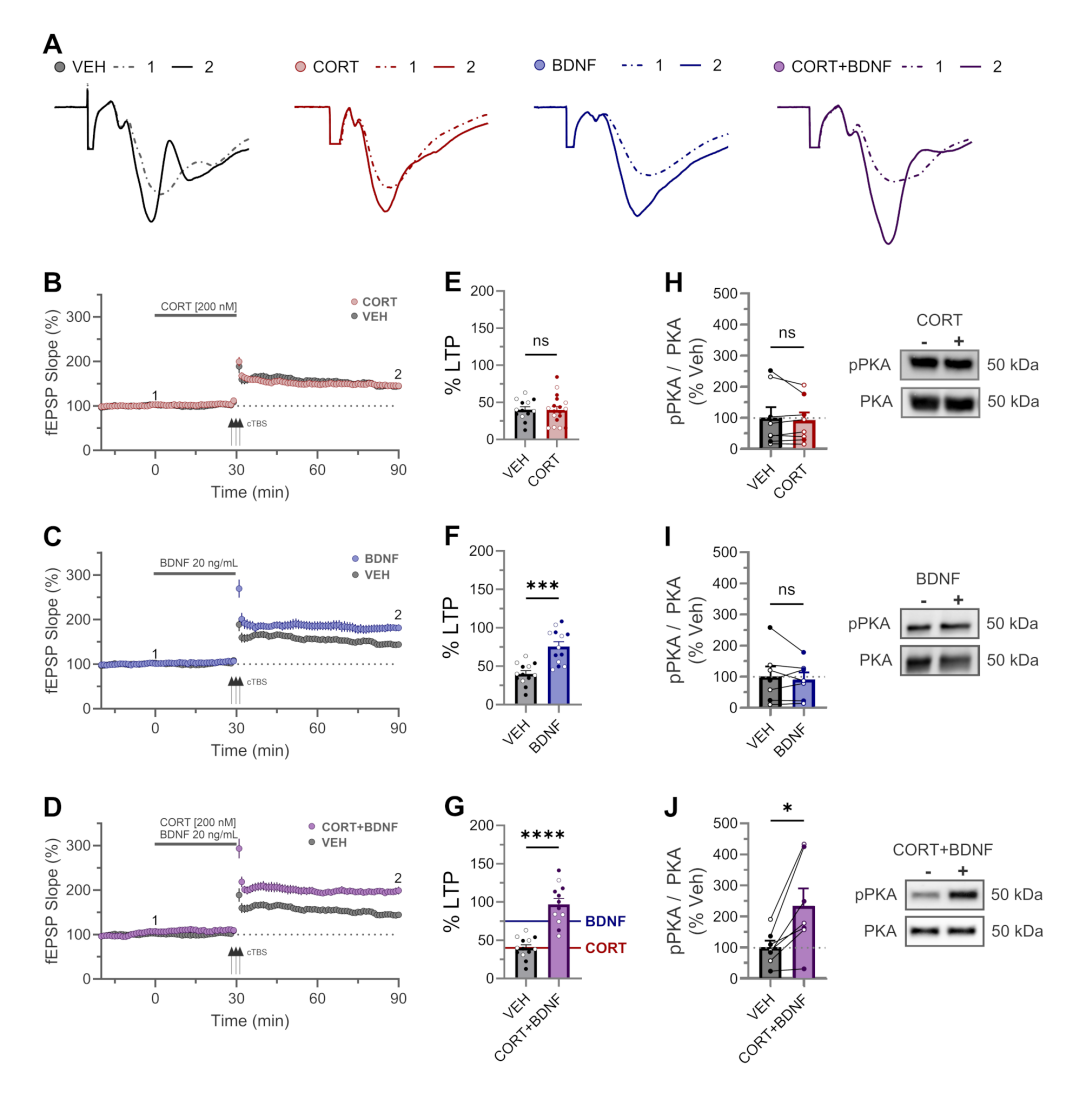
Enhanced LTP and phosphorylation of PKA following combined application of CORT and BDNF. **(A)** CA3-CA1 5-min average fEPSP synaptic traces from representative CTRL, CORT, BDNF, CORT + BDNF experiments (B-D, respectively) sampled at baseline ① (dashed line) and 60 min after cTBS induction ② (solid line). **(B-D)** Time course of synaptic responses for each of CORT **(B)**, BDNF **(C)**, and CORT + BDNF **(D)**. Three black arrows represent the cTBS conditioning stimulus. Solid grey bar represents compound (as specified) wash-on period (30 min). **(E-G)** LTP quantification revealed significant LTP enhancement using a one way-ANOVA (F(3,52) = 20.68, *p* = 0.0001) for BDNF (**F**; *p* = 0.0002) and CORT + BDNF (**G**; *p* < 0.0001) treatment but not CORT (**E**; *p* = 0.97) (female open circle, male closed circle). CORT + BDNF was significantly enhance compare to BDNF alone (**F vs. G**: mean difference = + 21 ± 8%, t(52) = 2.6, *p* = 0.01) **(H-J)** Exemplar western blots (right) and quantification (left) of phosphorylated PKA (T197) relative to total PKA levels (normalized to vehicle, VEH) after 30 min application of CORT (H), BDNF (I), or CORT + BDNF (J), respectively. CORT + BDNF was the only condition to display an increased pPKA signal (*p* = 0.01). The two lanes refer to VEH (-) versus compound (+) application. Connected lines refer to data from pairs of pooled hippocampal slices obtained from the same animal

It has been reported that the brief application of CORT results in enhanced LTP induced by a tetanus delivered to the SCC pathway [5]. We confirmed this observation using a high frequency stimulation (HFS) induction (Suppl. Figure 1), but to our surprise we saw no effect of CORT on LTP induced by TBS (Figs. 1A, B and E and 40 ± 5%, *n* = 18, t(52) = 0.04, *p* = 0.99, d = 0.01). In contrast, BDNF resulted in enhanced TBS-induced LTP (Figs. 1A, C and F and 75 ± 6%, *n* = 13, t(52) = 4.4, *p* = 0.0002, d = 1.92), which is consistent with previous work [14]. Interestingly, when delivered together, the effect of CORT + BDNF (97 ± 8%, *n* = 12, t(52) = 6.9, *p* < 0.0001, d = 2.2) was significantly greater than that of BDNF alone (Fig. 1A, D, G; mean difference = + 21 ± 8%, t(52) = 2.6, *p* = 0.01, d = 0.66). There was no significant effect of sex on the outcome variable, as indicated by a non-significant row factor in our two-way ANOVA (F(1, 50) = 0.038, *p* = 0.8459), with a negligible contribution to total variance (0.03%), and a mean difference of -1.20 (± 6.14 SEM) between males (61.83) and females (63.03).

As an initial step to exploring the mechanism underlying the synergistic action of CORT + BDNF, we assessed PKA activation by measuring the T197 phosphorylation site of the catalytic subunit of PKA, which is essential for activity [15]. As compared to VEH, neither CORT (Fig. 1H) nor BDNF (Fig. 1I) applied alone affected PKA phosphorylation (CORT: 91 ± 25%, t(7) = 0.80, *p* = 0.45, d = 0.10; BDNF: 91 ± 22, t(6) = 0.67, *p* = 0.53, d = 0.13). In contrast, the combined application of CORT + BDNF robustly induced PKA phosphorylation (Figs. 1J and 234 ± 56%, t(6) = 3.5, *p* = 0.01, d = 1.21).

## Discussion

In the present study we have shown that CORT + BDNF facilitates LTP to a greater extent than BDNF alone. This is a noteworthy observation since both CORT and BDNF are released during exercise and so may together be important physiological mediators of exercise-enhanced cognition. Although our model is not able to fully recapitulate the nuanced patterns of release of these molecules in vivo surrounding a single session of exercise, these ex vivo experiments provide a foundation to which the potential synergistic relationship of these molecules on synaptic plasticity may be assessed; an area of potential future investigation as previous findings suggest BDNF perfusion rate can impact synaptic plasticity [4].

In terms of mechanism, it has been shown that stress enhances LTP by enabling the induction of LTP2 by a stimulus that ordinarily would only induce LTP1 [5]. A similar induction of LTP2 can be achieved by spaced episodes of tetanic or TBS protocols, where the first episode can be considered as priming for LTP2 [2]. In both cases the “priming” involves a PKA-dependent mechanism. Our observation that CORT + BDNF also activates PKA is consistent with this type of mechanism. Future experiments are required to test the sensitivity of the CORT + BDNF facilitation of LTP to PKA inhibitors. A couple of other questions warrant further investigation. Firstly, how do CORT and BDNF act synergistically? One possibility is that CORT drives PKA and BDNF drives PI3K and that these signaling pathways converge downstream of the receptors, potentially at the level of glycogen synthase kinase-3 (GSK-3). However, the observation that CORT alone did not activate PKA under the conditions of the present experiments suggests an additional convergence at the level of PKA activation. Secondly, why was CORT + BDNF required to facilitate TBS-induced LTP whereas, in agreement with previous work [5], CORT alone was sufficient to facilitate HFS-induced LTP? One possibility is that HFS generates a sufficiently large Ca^2+^ signal to activate PKA (via the Ca^2+^-sensitive adenylyl cyclases) without the requirement of the convergent signal initiated by BDNF.

We focused our investigation on the well-validated phosphorylation site T197 of the PKA catalytic subunit, which serves as a robust indicator of its activation state. However, PKA function is also regulated through multiple spatial mechanisms, including subcellular localization via AKAP scaffolding proteins and compartmentalized cAMP signaling. Future studies aimed at integrating these dimensions could provide further insight into how stress and neurotrophic signals converge to modulate PKA signaling with greater spatial and temporal precision.

In conclusion, we hypothesize that during exercise, BDNF and CORT act together to enable the induction of LTP2. These changes are predicted to enhance learning and memory by increasing the propensity of LTP2 across brain networks. Future studies will be aimed at testing this hypothesis during physical exercise.

## Electronic supplementary material

Below is the link to the electronic supplementary material.

Supplementary Material 1

## Data Availability

No datasets were generated or analysed during the current study.
