# Correction to: Synergistic actions of corticosterone and BDNF on rat hippocampal LTP

**DOI:** 10.1186/s13041-025-01234-6

**Published:** 2025-07-24

**Authors:** Jonathan S. Thacker, Liam T. Ralph, Laura Koek, Aram Abbasian, Luis B. Bettio, Ashleigh E. Smith, John Georgiou, Brian R. Christie, Graham L. Collingridge

**Affiliations:** 1 Lunenfeld-Tanenbaum Research Institute, Mount Sinai Hospital, Sinai Health, Toronto, ON M5G 1X5 Canada; 2 Tanz Centre for Research in Neurodegenerative Diseases, Temerty Faculty of Medicine, University of Toronto, Toronto, ON Canada; 3 Department of Physiology, Temerty Faculty of Medicine, University of Toronto, Toronto, ON Canada; 4 Division of Medical Sciences, University of Victoria, Victoria, BC Canada; 5 Alliance for Research in Exercise Nutrition and Activity (ARENA) Research Group, Division of Health Sciences, University of South Australia, Adelaide, Australia; 6 Behaviour, Brain, and Body (BBB) Research Group, Division of Health Science, University of South Australia, Adelaide, Australia; 7 Island Medical Program, University of British Columbia, Victoria, BC Canada

**Correction to: Mol Brain 18, 42 (2025)**

10.1186/s13041-025-01213-x

Following publication of the original article [1], the authors identified that Fig. 1 was of poor quality while source Fig. 1 was of high quality. An error was made in the author name Ashleigh E Smith as it should have a middle initial ‘E’ instead ‘S’.

The incorrect Fig. 1:

**Fig. 1:**
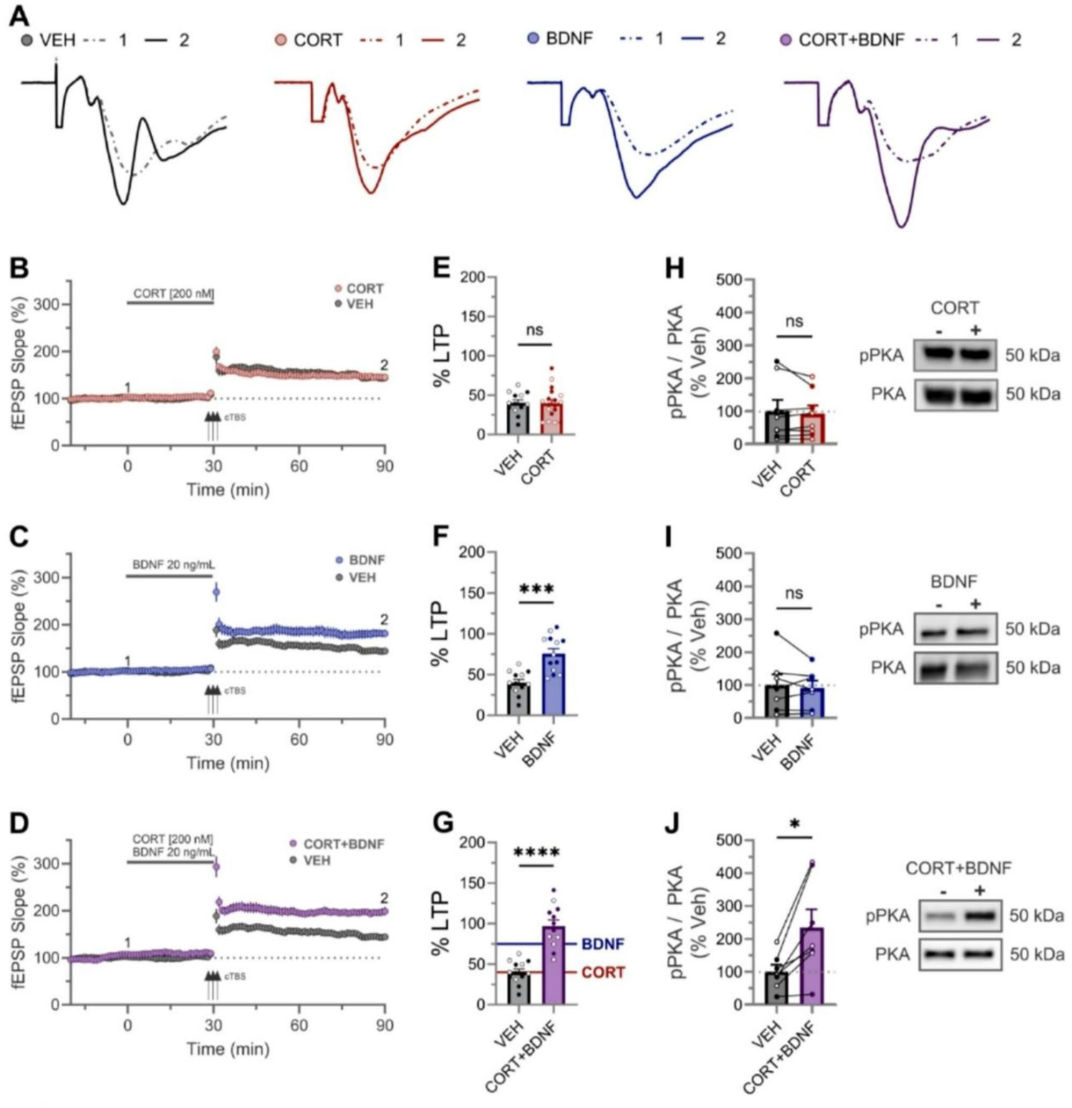
Enhanced LTP and phosphorylation of PKA following combined application of CORT and BDNF. **(A)** CA3-CA1 5-min average fEPSP synaptic traces from representative CTRL, CORT, BDNF, CORT + BDNF experiments (B-D, respectively) sampled at baseline ① (dashed line) and 60 min after cTBS induc-tion ② (solid line). **(B-D)**Time course of synaptic responses for each of CORT **(B)**, BDNF **(C)**, and CORT + BDNF **(D)**. Three black arrows represent the cTBS conditioning stimulus. Solid grey bar represents compound (as specified) wash-on period (30 min). **(E-G)** LTP quantification revealed significant LTP enhancement using a one way-ANOVA (F(3,52) = 20.68, *p* = 0.0001) for BDNF (**F**; *p* = 0.0002) and CORT + BDNF (**G**; *p* < 0.0001) treatment but not CORT (**E**; *p* = 0.97) (female open circle, male closed circle). CORT + BDNF was significantly enhance compare to BDNF alone (**F vs. G**: mean difference = + 21 ± 8%, t(52) = 2.6, *p* = 0.01) **(H-J)** Exemplar western blots (right) and quantification (left) of phosphorylated PKA (T197) relative to total PKA levels (normalized to vehicle, VEH) after 30 min application of CORT (H), BDNF (I), or CORT + BDNF (J), respectively. CORT + BDNF was the only condition to display an increased pPKA signal (*p* = 0.01). The two lanes refer to VEH (-) versus compound (+) application. Connected lines refer to data from pairs of pooled hippocampal slices obtained from the same animal

The correct Fig. 1:

**Fig. 1:**
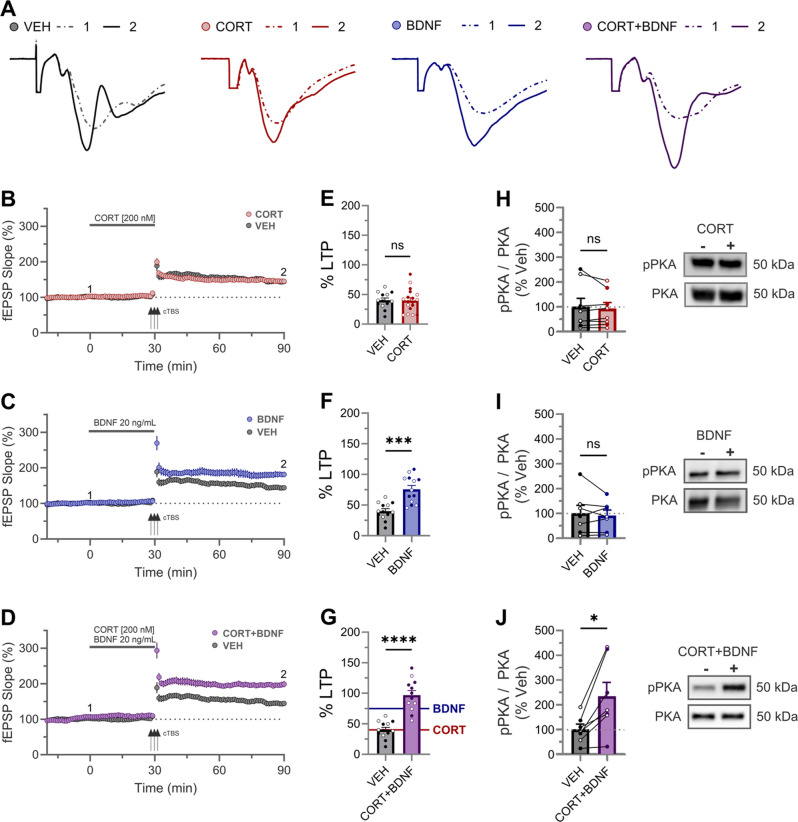
Enhanced LTP and phosphorylation of PKA following combined application of CORT and BDNF. **(A)** CA3-CA1 5-min average fEPSP synaptic traces from representative CTRL, CORT, BDNF, CORT + BDNF experiments (B-D, respectively) sampled at baseline ① (dashed line) and 60 min after cTBS induc-tion ② (solid line). **(B-D)**Time course of synaptic responses for each of CORT **(B)**, BDNF **(C)**, and CORT + BDNF **(D)**. Three black arrows represent the cTBS conditioning stimulus. Solid grey bar represents compound (as specified) wash-on period (30 min). **(E-G)** LTP quantification revealed significant LTP enhancement using a one way-ANOVA (F(3,52) = 20.68, *p* = 0.0001) for BDNF (**F**; *p* = 0.0002) and CORT + BDNF (**G**; *p* < 0.0001) treatment but not CORT (**E**; *p* = 0.97) (female open circle, male closed circle). CORT + BDNF was significantly enhance compare to BDNF alone (**F vs. G**: mean difference = + 21 ± 8%, t(52) = 2.6, *p* = 0.01) **(H-J)** Exemplar western blots (right) and quantification (left) of phosphorylated PKA (T197) relative to total PKA levels (normalized to vehicle, VEH) after 30 min application of CORT (H), BDNF (I), or CORT + BDNF (J), respectively. CORT + BDNF was the only condition to display an increased pPKA signal (*p* = 0.01). The two lanes refer to VEH (-) versus compound (+) application. Connected lines refer to data from pairs of pooled hippocampal slices obtained from the same animal

The incorrect author name reads:

Ashleigh S. Smith.

The correct author name should read:

Ashleigh E. Smith.

The initial of the given author and Fig. 1 have been updated and the original article [1] has been corrected.
