# Supplementary material for: Synergistic actions of corticosterone and BDNF on rat hippocampal LTP
Source: Mol Brain. 2025 May 12;18:42. doi: 10.1186/s13041-025-01213-x (PMC12070750; doi:10.1186/s13041-025-01213-x)
Supplement: Supplementary file 1 — Supplementary Material 1 [file 13041_2025_1213_MOESM1_ESM.docx]

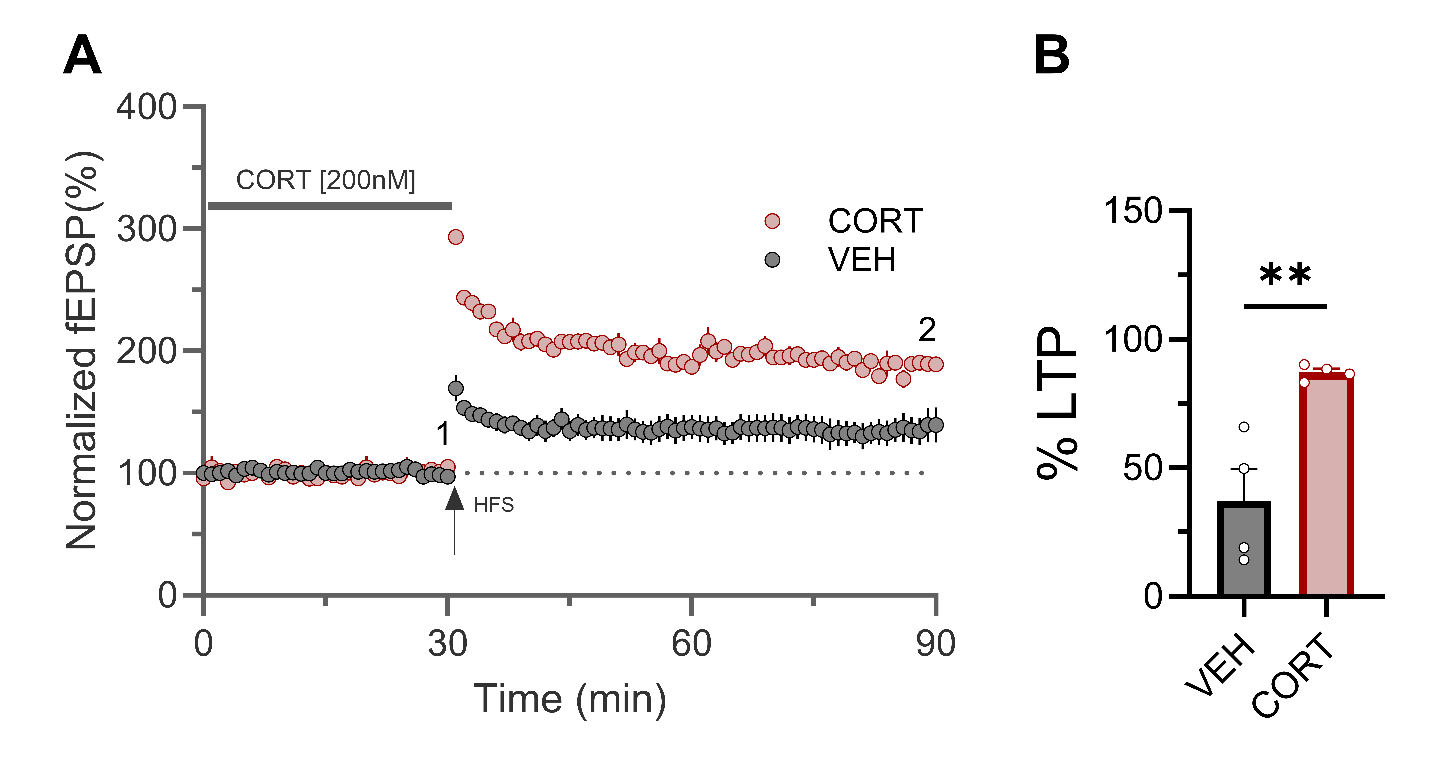


***Supplemental Figure 1***: **Effect of CORT on HFS-induced LTP in the CA1 of the rat hippocampus.** **(A)** Time course experiment plotting fEPSP slope for each of VEH and CORT (superimposed) normalized to corresponding baseline. Black arrow represents timing of the HFS conditioning stimulus delivery. Solid grey bar represents CORT wash-on period (30 min). **(B)** LTP quantification revealed significant enhancement for CORT over VEH (p = 0.007). LTP calculated as fEPSP slope change between baseline ①and 60 min after HFS induction ②.
